# Epsilonproteobacteria in Humans, New Zealand

**DOI:** 10.3201/eid1803.110875

**Published:** 2012-03

**Authors:** Angela J. Cornelius, Stephen Chambers, John Aitken, Stephanie M. Brandt, Beverley Horn, Stephen L.W. On

**Affiliations:** Institute of Environmental Science and Research, Christchurch, New Zealand (A.J. Cornelius, S.M. Brandt, B. Horn, S.L.W. On);; Christchurch School of Medicine, Christchurch (S.T. Chambers);; Southern Community Laboratories, Christchurch (J. Aitken)

**Keywords:** Campylobacter, Helicobacter, Arcobacter, PCR, epidemiology, volunteers, diarrhea, molecular, diagnostic, bacteria, New Zealand

## Abstract

Using PCR–denaturing gradient gel electrophoresis, we examined 49 fecal samples from healthy volunteers and 128 diarrhea specimens to assess the distribution of Epsilonproteobacteria that might be routinely overlooked. Our results suggest that certain taxa that are not routinely examined for could account for a proportion of diarrhea of previously unknown etiology.

Acute gastrointestinal illness is a major health concern in industrialized countries. In New Zealand, an estimated 4.6 million cases of acute gastrointestinal illness occur every year (*1*). For many known causes of acute gastrointestinal illness, conventional methods of diagnosis are available; yet, ≈80% of diarrhea cases go undiagnosed (*1**,**2*). This lack of data concerning causes of diarrhea hinders the development of intervention strategies.

The class Epsilonproteobacteria is a distinct, diverse bacterial group containing ≈100 taxa (*3*), including *Campylobacter jejuni,* recognized as the most frequent bacterial cause of human gastroenteritis worldwide (*4**,**5*). Many other epsilonproteobacterial species have been associated with diarrhea, but accurate estimates of the prevalence and role of individual species and proof of a primary pathogenic role have been elusive. Methods commonly used for isolating *C. jejuni* are not well suited for many other species, and the complex taxonomy of the group makes identification difficult (*4*). Nevertheless, the body of evidence supporting a causative role for several taxa has grown (*5**–**7*). During September 2007–June 2009, we examined fecal samples from healthy volunteers and from patients with diarrhea in New Zealand by using a PCR–denaturing gradient gel electrophoresis (DGGE) method shown to detect and identify Epsilonproteobacteria (*8*).

## The Study

Healthy volunteers were recruited during 2 separate periods in September 2007 and June 2009. The first recruitment period (18 specimens) did not specifically exclude volunteers who had had gastrointestinal disturbances in the 10 days before sampling. The second (31 specimens) were healthy volunteers who had normal bowel habit, no diarrheal disease for >6 weeks, no antimicrobial drug therapy for >4 weeks, and no medication except for asthma inhalers or antihypertensive or contraceptive medication. Volunteers defecated into a bottle suspended in the lavatory bowl with tissue paper to prevent it falling into the water. The Upper South A Regional Ethics Committee (Christchurch, New Zealand) and the multi-ethics committee of the Ministry of Health, New Zealand (MEC/08/52/EXP), granted ethics approval for the study.

Diarrhea specimens (submitted without patient details during 2008) were distributed among 3 categories, as follows. First were 32 samples in which no causal agent was found; pathogens were excluded by routine examination with conventional diagnostic techniques for bacteria, parasites, and norovirus at Southern Community Laboratories. Second were 57 samples in which a specific causal agent was not found; samples were examined at the Institute of Environmental Science and Research (ESR, Christchurch, New Zealand) reference laboratory by using conventional methods for a specific pathogen at the request of the submitting laboratory. Third were 39 samples in which a known gastrointestinal pathogen had been detected at ESR.

Samples were refrigerated for 24–48 h before DNA extracts were prepared by using the revised protocol described in the ZR Fecal DNA Kit (Zymo Research, Irvine, CA, USA). Fecal DNA extracts were examined with the PCR-DGGE for Epsilonproteobacteria as described (*8*). After visualization of the PCR-amplified product, individual DNA bands were excised and then DNA was eluted by diffusion into buffer and reexamined by PCR to obtain partial 16S rDNA amplicons for sequencing. Sequences were edited (primer sequences were removed) and subsequently compared with those in GenBank by using BLAST (http://blast.ncbi.nlm.nih.gov/Blast.cgi). Comparisons were made during April 2011. Assignment of sequences to a taxon was based on the E (expect) values obtained and on expert opinion of the taxonomic distance between the most likely matches obtained. BLAST matches yielded E-values ranging from 7.13e-62 to 2.26e-124.

Of 177 samples from the healthy volunteers and patients with diarrhea, 159 contained Epsilonproteobacteria, of which 20 contained >1 taxa (Table). *C. rectus/showae*, *C. sputorum*, *C. upsaliensis*, *Helicobacter* *pullorum*, and *H. pylori/heilmannii/nemestrinae* were detected in 11 (8.6%) of the 128 diarrhea samples but not in fecal specimens from volunteers. *Cryptosporidium* spp. also were present in 2 diarrhea samples in which *C. rectus/showae* were detected. In addition, norovirus was detected in the *C. sputorum*–positive sample. *C. curvus* and *C. jejuni/coli* were found in diarrhea samples examined previously for specific pathogens only, as well as in 1 and 4 samples, respectively, from human volunteers. Sequences of the *C. concisus* complex, *C. ureolyticus*, *C. hominis*, and *C. gracilis* occurred frequently in samples from all study participants.

**Table:** Prevalence and distribution of the Epsilonproteobacteria taxa in fecal samples from 49 healthy volunteers and 128 persons with diarrhea, New Zealand*

Taxa	SCL	ESR−	ESR+	Vol
*Campylobacter jejuni/coli* complex	0	3	0	4
*C. ureolyticus*	3	10	1	12
*C. concisus*	17	27	16	26
*C. curvus*	0	1	0	1
*C. gracilis*	4	10	4	3
*C. hominis*	4	6	1	8
*C. rectus/showae*	2	1	2	0
*C. sputorum*	0	0	1	0
*Helicobacter* sp.	1	0	0	0
*C. upsaliensis/helveticus*	0	2	0	0
*H. pullorum*	0	2	0	0
No Epsiloproteobacterium	6	3	8	1

*Detected by PCR–denaturing gradient gel electrophoresis. SCL, samples examined by Southern Community Laboratories (Christchurch, New Zealand) found negative for all common pathogens; ESR–, diarrhea samples screened for specific pathogens by the Institute of Environmental Science and Research (Christchurch) at the request of the submitting laboratory and found negative; ESR+, diarrhea samples screened for specific pathogens by the Institute of Environmental Science and Research at the request of the submitting laboratory and found positive; vol, samples from volunteers with no known recent history of gastrointestinal illness. Specific pathogens found in ESR+ samples included *Cryptosporidium* spp., *Giardia* spp., norovirus, *Bacillus cereus*, toxigenic *Staphylococcus aureus*, and toxigenic *Clostridium perfringens*.

We used χ^2^ analysis to determine whether the proportion of the 32 diarrhea samples subjected to a complete pathogen screen differed significantly from fecal samples from the second group of 31 volunteers in which these organisms were detected. The pathogen screen contained *C.* (*Bacteroides*) *ureolyticus*, *C. concisus* complex, *C. hominis*, or *C. gracilis*. We found no statistical difference between the proportions detected in these 2 groups of samples.

## Conclusions

Although many species belonging to the Epsilonproteobacteria have been associated with gastrointestinal illness for decades, few are proven primary pathogens. By using PCR-DGGE to examine feces from healthy volunteers and patients with diarrhea, we aimed to indicate which taxa might be present as commensal flora and which might have a causal role. *C. upsaliensis/helveticus*, *H. pullorum*, *H. pylori/heilmannii/nemestrinae* were all detected in diarrhea specimens but not in specimens from healthy volunteers; no other pathogen was found in these diarrhea specimens. *C. upsaliensis* is presumed to be pathogenic (*7*). *H. pullorum* is poorly studied but bears sufficient similarity to diarrheogenic *C. jejuni* at the molecular–genetic level (*9*) to support a causative role in gastrointestinal disease, at least for some strains. Poultry harbor *H. pullorum* (*10*), and thus represent a vector for foodborne transmission. Use of the PCR-DGGE method (*8*) on domestic drinking and commercial scald water used in New Zealand chicken production detected *H. pullorum* in 2 of 5 samples tested (data not shown). Although detection of the taxa *H. pylori/heilmannii/nemestrinae* might simply represent gastric carriage (the natural environment for these species), perhaps gastric disturbances result in diarrheal sequalae. Even though *C. rectus/showae* were also detected only in diarrhea samples, 2 specimens also harbored *Cryptosporidium* spp. In addition, norovirus was detected in the diarrhea sample in which *C. sputorum* was found.

We detected *C. jejuni/coli* in 3 samples examined for, but not containing, *E. coli* O157, which indicates that some cases of campylobacteriosis go undiagnosed. To our surprise, we detected *C. jejuni/coli* in several fecal samples from healthy volunteers. This detection might represent asymptomatic carriage of *C. jejuni/coli,* a phenomenon more commonly observed in developing countries where repeated exposure during a prolonged period results in tolerance (*11*). The high incidence of infection in New Zealand makes this hypothesis credible.

*C. concisus* was the most frequently encountered species in this study and occurred in participants from both groups. Strains identified as *C. concisus* with conventional methods might belong to genetically distinct but phenotypically indistinguishable genomospecies differing in their pathogenic potential (*12*). The PCR-DGGE used here cannot differentiate *C. concisus* genomospecies; thus strains detected in volunteers and strains found in diarrhea samples might represent distinct genomospecies with different pathogenic potentials.

We detected *C. hominis*, *C. gracilis*, and *C. ureolyticus* in fecal samples of healthy volunteers and patients with diarrhea. *C. hominis* has long been considered a commensal (*13*). A molecular study found *C. ureolyticus* in 83 (23.8%) of 349 *Campylobacter* spp.–positive diarrhea samples, but no healthy controls were examined (*14*). Our data suggest these species are unlikely causes of diarrhea.

Our results indicate that certain Epsilonproteobacteria that are not routinely examined for account for a proportion of diarrhea cases of previously unknown etiology. PCR-DGGE is a useful tool to study the prevalence and distribution of these bacteria. *C. concisus* genomospecies are frequently detected in human disease (*5*,*15*; this study); elucidation of their pathogenicity should be considered a public health research issue.
